# Supplementary material for: Reduced proteasome activity in the aging brain results in ribosome stoichiometry loss and aggregation
Source: Mol Syst Biol. 2020 Jun 18;16(6):e9596. doi: 10.15252/msb.20209596 (PMC7301280; doi:10.15252/msb.20209596)
Supplement: Supplementary file 1 — Expanded View Figures PDF [file MSB-16-e9596-s001.pdf]

# Expanded View Figures

**Figure EV1. Proteome analysis of killifish brain aging and comparison of transcriptome dataset.**

A Principal component analysis (PCA) of brain samples based on proteome profiles obtained by tandem mass tag (TMT) quantification for the 12 vs 5 wph and 39 vs 12 wph comparisons. For each sample group, the four samples displaying the highest within group correlation were selected and used for differential expression analysis. The smaller dots represent individual samples and the larger dots the centroids of each age-matched group. Ellipses represent 95% confidence intervals. The percentage of variance explained by the first two PC axes is reported in the axis titles.

B Overlap between the quantified protein groups across the two TMT experiments. Only proteins quantified with at least two unique (proteotypic) peptides were considered.

C Barplots representing enriched KEGG pathways among proteins affected by aging in killifish brain. Pathway enrichment was performed using *gage* (Luo *et al*, 2009). Significant pathways enriched among up-regulated (red) or down-regulated proteins (blue) are shown ($P < 0.05$). The complete list of enriched pathways is reported in Dataset EV2.

D Principal component analysis (PCA) of brain samples based on total RNA ("this study", same sample used for proteome analysis) or polyA$^{+}$ RNA sequencing (Baumgart *et al*, 2014)). The smaller dots represent individual samples and the larger dots the centroids of each age-matched group. Ellipses represent 95% confidence intervals. The percentage of variance explained by the first two PC axes is reported in the axis titles.

E Correlation between transcript and protein during aging. RPKM and iBAQ values were used to estimate transcript and protein levels from RNA-seq (Baumgart *et al*, 2014) and TMT-based proteomics data (this study). Since in this case samples were not matched, proteomic data from each individual sample were compared against combined RPKM values obtained from the average of the samples for each group. The ANOVA test was performed to evaluate significance among the age groups (mean correlation at 5 wph: 0.43; at 12 wph: 0.38; and at 39 wph: 0.32; $P = 2.02e{-12}$, $n = 5$ per age group). In boxplots, the horizontal line represents the median, the bottom, and top of the box the 25$^{th}$ and 75$^{th}$ percentile, respectively, and the whiskers extend 1.5-fold the interquartile range.

F Comparison between RNA-seq data obtained in this work and previous data obtained from (Baumgart *et al*, 2014). Fold changes ($\log_2$) were compared and plotted for both the aging comparisons (12 wph vs 5 wph and 39 wph vs 12 wph) for significantly affected transcripts in both comparisons (adj. $P < 0.01$).

G Statistics of differentially expressed genes in the two RNA-seq datasets. The same datasets used for G were analyzed. Differentially expressed genes obtained for three different age comparisons were compared between the datasets. Dark gray boxes correspond to not-affected transcripts, while yellow (adj. $P < 0.01$) and orange (adj. $P < 0.01$ and absolute $\log_2$ fold change $> 0.5$) boxes to differentially expressed genes. The complete list of quantified transcripts is reported in Dataset EV3.

© The Authors

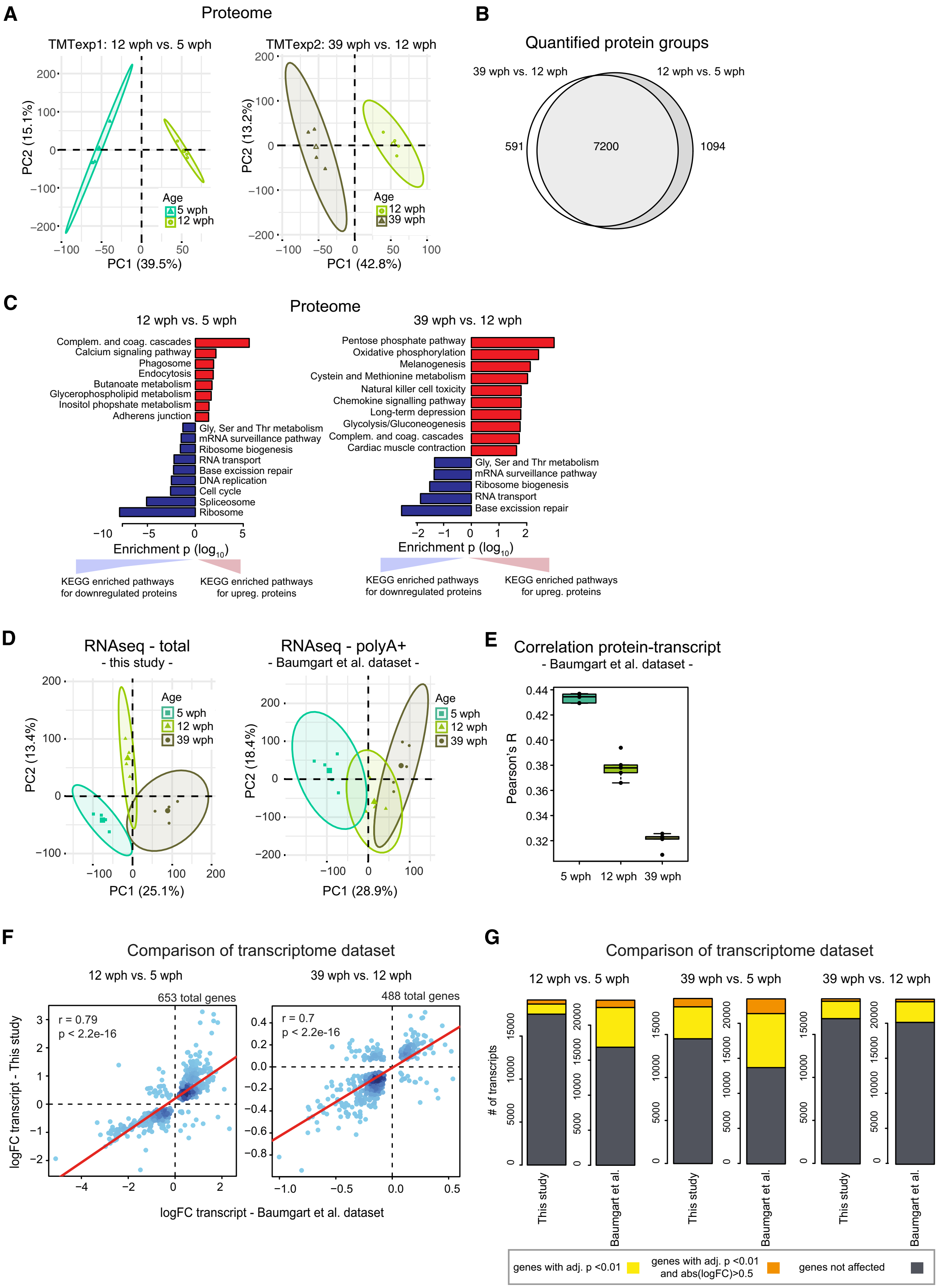


**Figure EV1.**

 ©2020 The Authors

**Figure EV2. Validation of discordant transcript and protein changes during aging.**

A Analysis of age-related changes of abundance for long-lived proteins. Average fold changes ($log_2$) are displayed as bars for transcripts (light blue) and proteins (dark blue), for a subset of extremely long-lived proteins identified by (Toyama *et al*, 2013).

B Validation of an increased level of glial fibrillary acidic protein (GFAP) in old killifish brain that manifests exclusively at the protein level. Double immunostaining for S100 (green) and GFAP (red) in the central region of the optic tectum of 7 wph fish (upper panels) vs 30 wph fish (lower panels). Scale bar = 100 μm. Average transcript and protein fold changes ($log_2$) are displayed as bars for transcript (light blue) and protein (dark blue) levels of GFAP.

C Age-related changes of markers for specific cell types in the brain of *Nothobranchius furzeri*. Fold changes for different age pairwise comparisons (12 vs 5 wph in white; 39 vs 5 wph in blue) were plotted using the cell-type markers from (Sharma *et al*, 2015) (in particular neuronal, oligodendrocytes, microglia, and astrocytes markers). For each cell type, the top 100 marker genes (ranked according to adj. *P* value) were plotted. Both transcriptome and proteome fold changes are displayed. In boxplots, the horizontal line represents the median, the bottom, and top of the box the $25^{th}$ and $75^{th}$ percentile, respectively, and the whiskers extend 1.5-fold the interquartile range.

D Workflow for the identification of proteins potentially affected by miRNA regulation during aging. For this analysis, proteins were divided into two groups: (i) proteins affected by aging (adj. $P < 0.05$) whose abundance change could be explained by transcript expression change and (ii) proteins affected by aging whose transcript was either regulated but with opposite fold change (discordant cases) or not regulated at all. miRNA analysis was performed only on the latter. To obtain a list of miRNAs affected by aging, differential expression was performed on both the age comparisons (12 vs 5 wph and 39 vs 12 wph) separately. The resulting list of miRNAs affected by aging is available in Dataset EV3.

© The Authors 

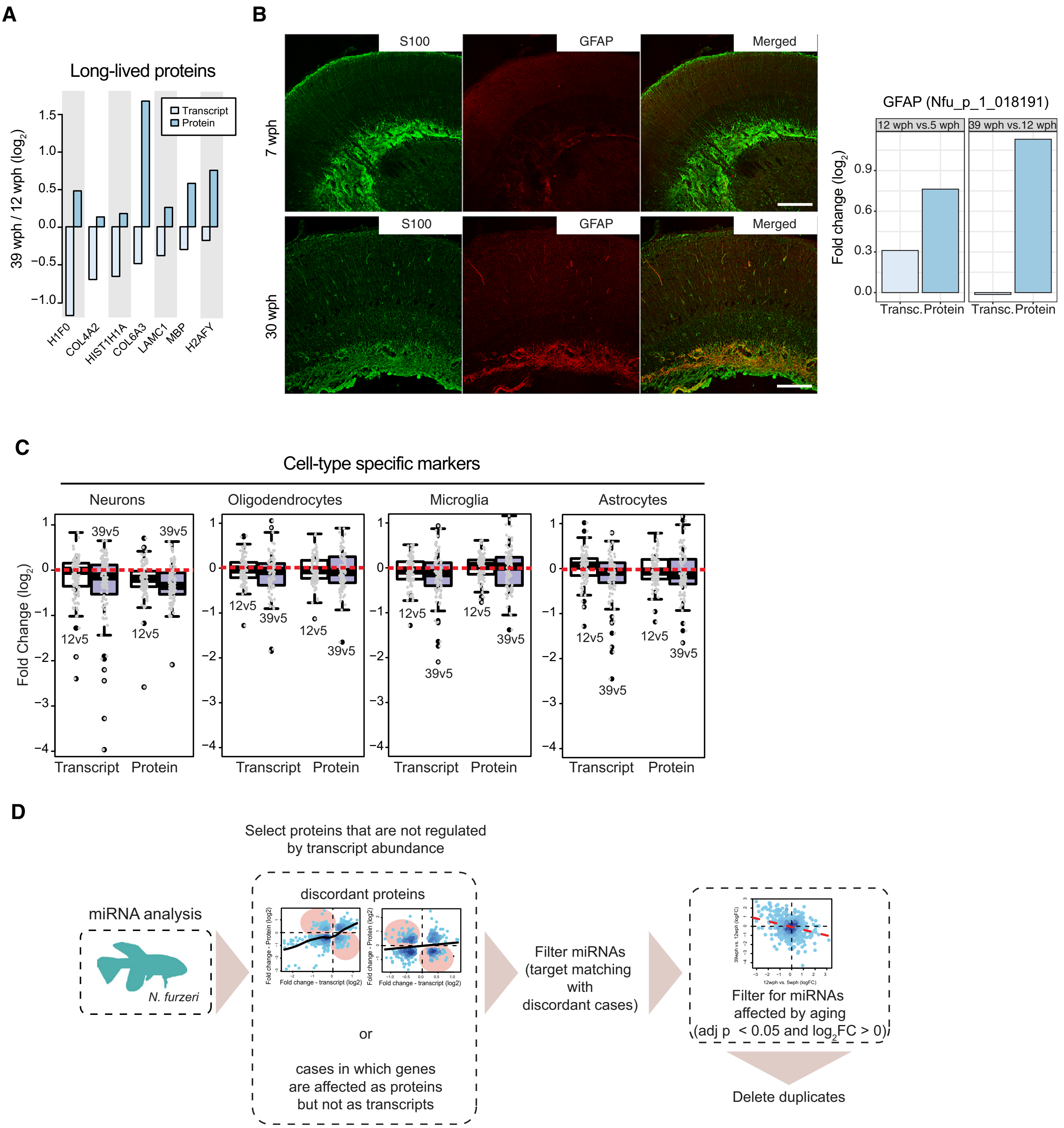


**Figure EV2.**

 ©2020 The Authors

**Figure EV3. Detailed view of age-related abundance changes for ribosomal transcripts and proteins.**

A, B Heatmap showing transcript and protein fold changes for members of the cytoplasmic (A) and mitochondrial (B) ribosome. Genes are annotated according to whether they are significantly affected at the transcript (adj. $P < 0.05$, black and white heatmap) or protein (adj. $P < 0.05$, black and green heatmap) level.

C Ribosome footprinting analysis of young and old killifish brain. Ribosome footprinting was performed from young (6 wph) and old (26 wph) killifish brains ($n = 4$ per age group). Fold changes were estimated for each gene from mean TPMs of young and old samples. For comparison, age-related changes in translation output measured in rat brain from (Ori *et al*, 2015) are shown. The central line of the violin plots indicates the median value.

D Statistics of members of protein complexes undergoing stoichiometry changes with aging. Members were considered as affected when their abundance differed significantly (adj. $P < 0.05$) from the mean of the protein complex to which they belong, as described in Ori *et al* (2016). Only protein complexes that had at least five members quantified were considered for each comparison. The complete list of stoichiometry changes is available in Dataset EV6.

E Degree of stoichiometry loss across different age comparisons. The interquartile range (IQR) of fold changes for members of the same protein complex was used to estimate the degree of stoichiometry loss (Janssens *et al*, 2015). All the measurements were performed in a single TMT experiment ($n = 3$ for each age group), and $P$ values were calculated using Wilcoxon rank-sum test. In boxplots, the horizontal line represents the median, the bottom, and top of the box the 25$^{th}$ and 75$^{th}$ percentile, respectively, and the whiskers extend 1.5-fold the interquartile range.

F Barplot showing the ranking of protein complexes based on the difference in protein level IQR for each complex between the 39 vs 12 wph and 12 vs 5 wph comparison. Selected complexes are highlighted and the percent of IQR increase between the two age comparisons is indicated in brackets.

© The Authors

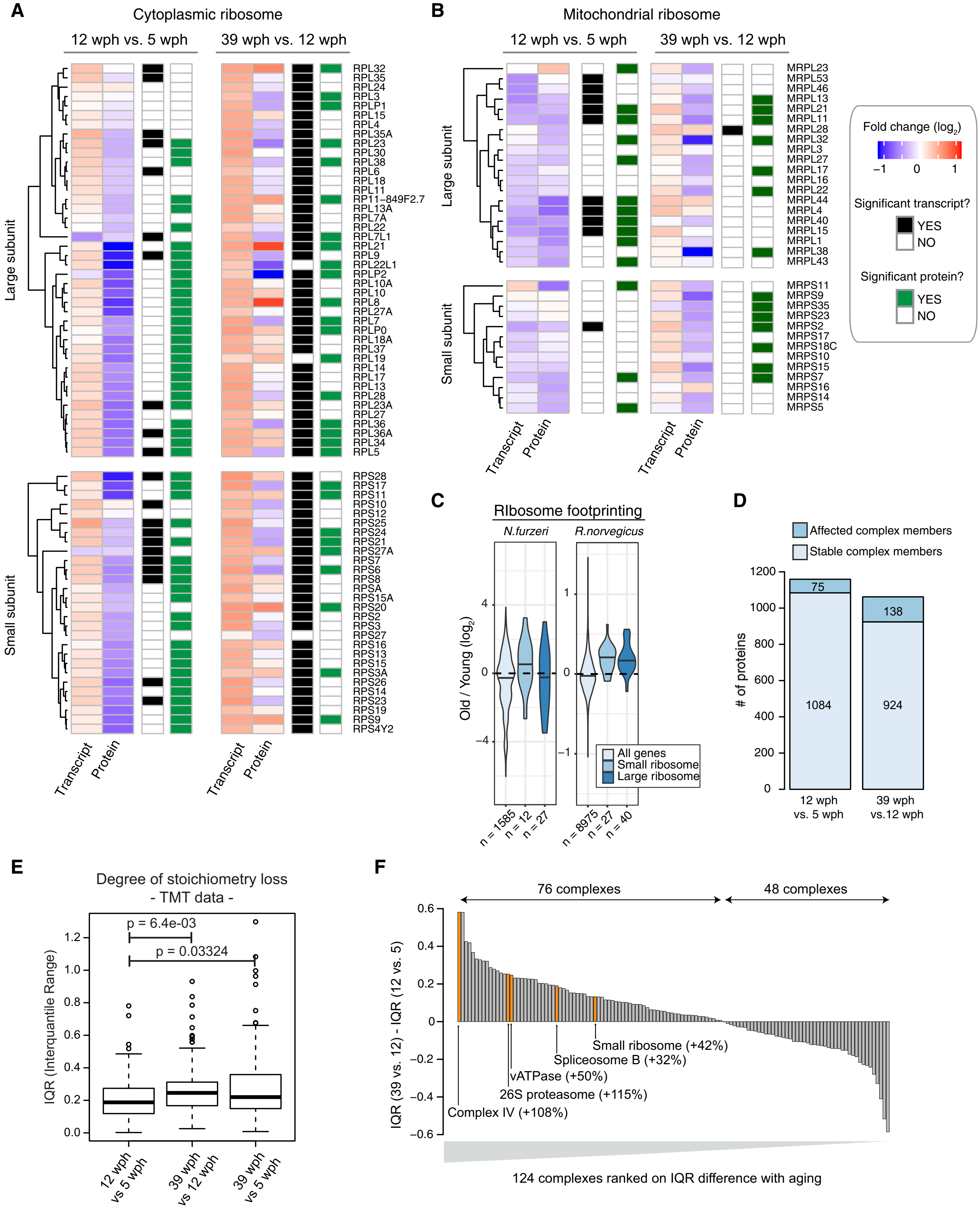


**Figure EV3.**

 ©2020 The Authors

**Figure EV4. Biophysical properties of protein enriched in protein aggregates and validation of ribosome aggregation by immunofluorescence in old killifish brain.**

A Workflow for the isolation of SDS-insoluble aggregates from mouse brain lysates.

B Coomassie-stained SDS–PAGE gel used for quantification of SDS-insoluble aggregates obtained from young and old mouse brain shown in Fig 3B. For each sample, a region of interest of the same area was selected for densitometry quantification (highlighted in yellow).

C Plot representing the fold enrichment in the insoluble fraction of protein with either high (top 20% scores) or low (bottom 20% scores) content of intrinsically disordered region, as predicted with the cleverSuite classifier. Violin plots: The solid line shows the median and the dotted lines the interquartile ranges. *$P < 0.05$, Kolmogorov–Smirnov test.

D Boxplots of complexes ranked according to the median enrichment of protein complex members in the aggregates ($n$ = 3 per group). Only complexes with at least 3 members quantified were considered. In boxplots, the horizontal line represents the median, the bottom, and top of the box the 25$^{th}$ and 75$^{th}$ percentile, respectively, and the whiskers extend 1.5-fold the interquartile range.

E–G Double labeling of telencephalic sections of *Nothobranchius furzeri* with Proteostat as a marker of aggregated proteins (red) and anti-LAMP1 as lysosomal marker (E and F, green), or RPS6 to evidence the ribosomal component of protein aggregates in old fish (G, green). Nuclear counterstaining was performed with DAPI (blue). The region of interest shown in Fig 3E and in (H) is indicated by a white box. Scale bars = 20 μm.

H Magnification of the selected area in (F) showing a detail of the co-localization between lysosomal structures (green) and protein aggregates (red) in the old brain. Scale bar = 10 μm.

I Ribosomal proteins enriched in mouse aggregates can also be identified in *N. furzeri* aggregates. Volcano plot based on protein quantification by label-free mass spectrometry depicting the enrichment of specific proteins in protein aggregates in mice (same as shown in Fig 3D). The *x*-axis indicates the $\log_2$ ratio between protein abundance in aggregates (Pellet) and starting total homogenate (TH). The horizontal dashed line indicates a *P* value cut-off of 0.05 and vertical lines a $\log_2$ fold change cut-off of ± 0.5. Proteins identified in mouse and killifish aggregates are highlighted as green dots (the entire list of proteins identified in killifish aggregates with at least two unique peptides in at least one replicate is reported in Dataset EV7).

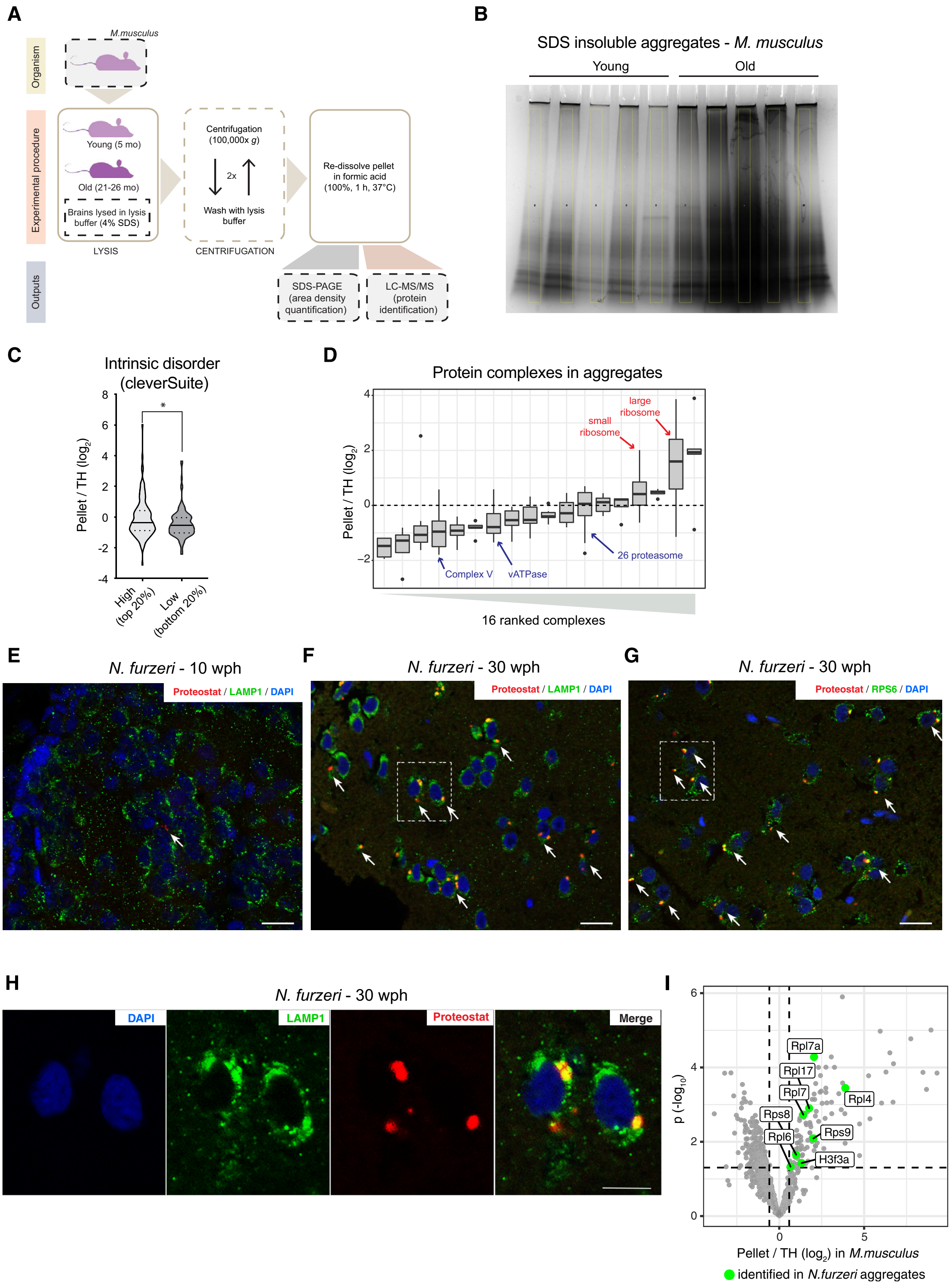


**Figure EV4.**



©2020 The Authors

**Figure EV5. Decreased proteasome activity in old killifish brain.**

A Immunoblot following native gel electrophoresis in brain extracts of killifish of different ages using an antibody recognizing the α subunits (1–7) of the proteasome.

B In-gel proteasome activity following native gel electrophoresis in brain extracts of killifish of different ages. This independent group of samples was quantified together with the ones displayed in Fig 4D, and the quantification results are displayed in Fig 4E.

C Low exposure of the blots shown in Figs 4D and EV5B. The loading of the native PAGE was based on protein quantification using the Bradford method, as described in Myeku *et al* (2011)

D Equal protein loading was confirmed by Ponceau S (bottom) and immunoblotting (top) against the housekeeping protein α-tubulin (T9026, Sigma, 1:5,000) using the same samples.

E Barplots depicting the quantification of chymotrypsin-like (CT-L) activity from native gels normalized to α-tubulin immunoblot. Activity of doubly capped (30S) or singly capped (26S) proteasomes is shown. $n \geq 5$ per sample group; error bars indicate standard error of the mean. *$P < 0.05$, **$P < 0.005$, one-way ANOVA, Holm–Sidak's multiple comparisons test. For each sample group, the mean value of activity in young samples (5 wph) was set to 100%.

© The Authors

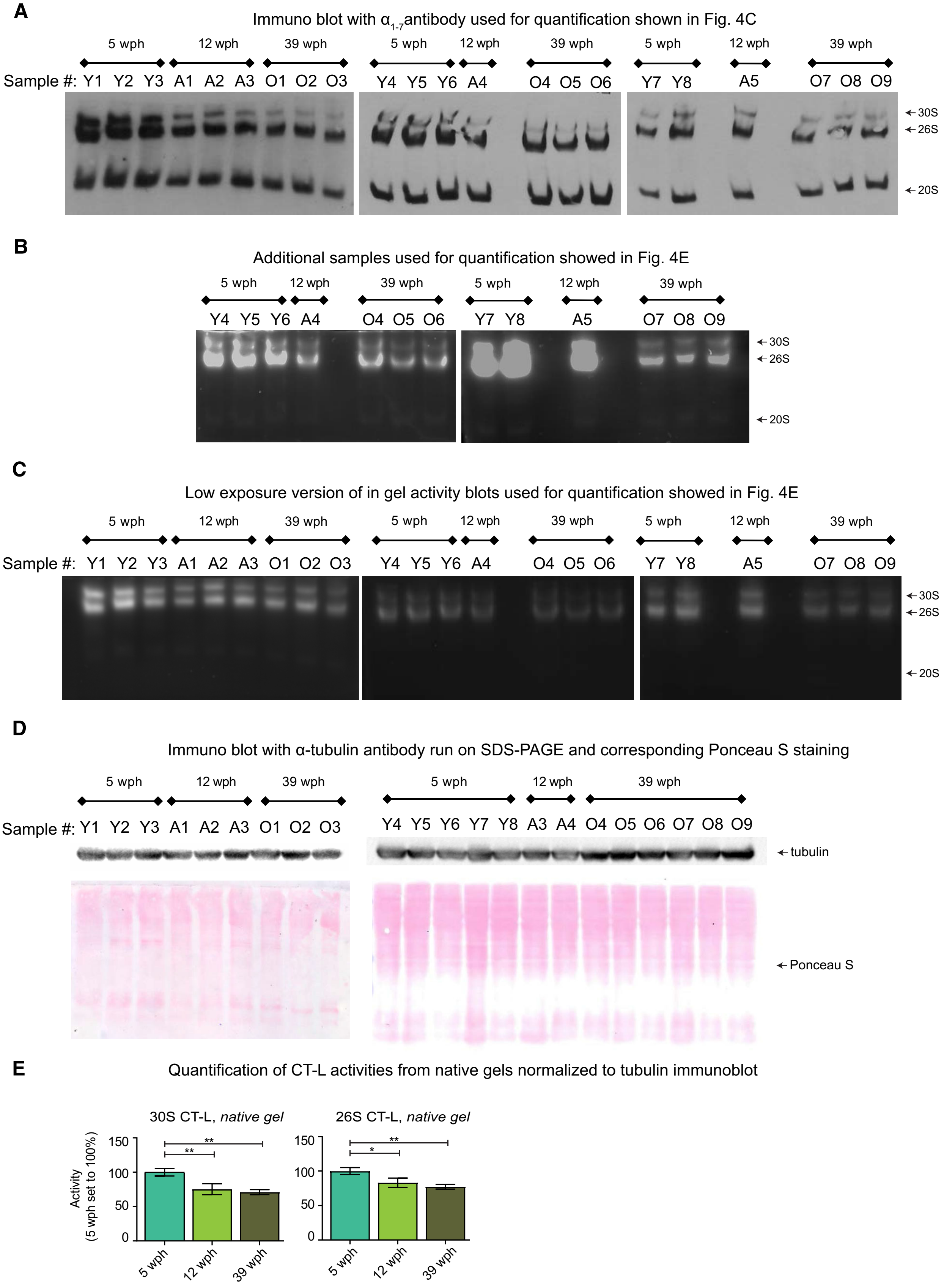
A
Immuno blot with α1-7antibody used for quantification shown in Fig. 4C
5 wph
12 wph
39 wph
Sample #: Y1 Y2 Y3 A1 A2 A3 O1 O2 O3 Y4 Y5 Y6 A4 O4 O5 O6 Y7 Y8 A5 O7 O8 O9
30S
26S
20S
B
Additional samples used for quantification showed in Fig. 4E
Y4 Y5 Y6 A4 O4 O5 O6 Y7 Y8 A5 O7 O8 O9
C
Low exposure version of in gel activity blots used for quantification showed in Fig. 4E
D
Immuno blot with α-tubulin antibody run on SDS-PAGE and corresponding Ponceau S staining
Y4 Y5 Y6 Y7 Y8 A3 A4 O4 O5 O6 O7 O8 O9
tubulin
Ponceau S
E
Quantification of CT-L activities from native gels normalized to tubulin immunoblot
30S CT-L, native gel
26S CT-L, native gel
Activity (5 wph set to 100%)
150
100
50
0
**
*
5 wph
12 wph
39 wph

Figure EV5.
