# Supplementary material for: Reduced proteasome activity in the aging brain results in ribosome stoichiometry loss and aggregation
Source: Mol Syst Biol. 2020 Jun 18;16(6):e9596. doi: 10.15252/msb.20209596 (PMC7301280; doi:10.15252/msb.20209596)
Supplement: Supplementary file 2 — Table EV1 [file MSB-16-e9596-s002.pdf]

**Table EV1 related to Figure 1.**

Statistics of transcriptome and proteome changes during *N. furzeri* brain aging.

| Protein groups | | | |
|---|---|---|---|
| | 12 vs. 5 stable | 12 vs. 5 up | 12 vs. 5 down |
| 39 vs. 12 stable | 3462 | 493 | 837 |
| 39 vs. 12 down | 690 | 111 | 252 |
| 39 vs. 12 up | 978 | 127 | 250 |

Out of 7200 detected proteins (overlap between the two conditions).

| Transcripts | | | |
|---|---|---|---|
| | 12 vs. 5 stable | 12 vs. 5 up | 12 vs. 5 down |
| 39 vs. 12 stable | 18557 | 605 | 244 |
| 39 vs. 12 down | 786 | 63 | 25 |
| 39 vs. 12 up | 885 | 144 | 92 |

Out of 21813 transcripts (overlap between the two conditions).

Legend

| |
|---|
| Stable = not significant |
| Down = adj. $p < 0.05$ & $\log_2$ FC $< 0$ |
| Up = adj. $p < 0.05$ & $\log_2$ FC $> 0$ |
